# *In Silico* Pooling of ChIP-seq Control Experiments

**DOI:** 10.1371/journal.pone.0109691

**Published:** 2014-11-07

**Authors:** Guannan Sun, Rajini Srinivasan, Camila Lopez-Anido, Holly A. Hung, John Svaren, Sündüz Keleş

**Affiliations:** 1 Department of Statistics, University of Wisconsin, Madison, Wisconsin, United States of America; 2 Waisman Center, Department of Comparative Biosciences, University of Wisconsin, Madison, Wisconsin, United States of America; 3 Department of Biostatistics and Medical Informatics, University of Wisconsin, Madison, Wisconsin, United States of America; Tel Aviv University, Israel

## Abstract

As next generation sequencing technologies are becoming more economical, large-scale ChIP-seq studies are enabling the investigation of the roles of transcription factor binding and epigenome on phenotypic variation. Studying such variation requires individual level ChIP-seq experiments. Standard designs for ChIP-seq experiments employ a paired control per ChIP-seq sample. Genomic coverage for control experiments is often sacrificed to increase the resources for ChIP samples. However, the quality of ChIP-enriched regions identifiable from a ChIP-seq experiment depends on the quality and the coverage of the control experiments. Insufficient coverage leads to loss of power in detecting enrichment. We investigate the effect of *in silico* pooling of control samples within multiple biological replicates, multiple treatment conditions, and multiple cell lines and tissues across multiple datasets with varying levels of genomic coverage. Our computational studies suggest guidelines for performing *in silico* pooling of control experiments. Using vast amounts of ENCODE data, we show that pairwise correlations between control samples originating from multiple biological replicates, treatments, and cell lines/tissues can be grouped into two classes representing whether or not *in silico* pooling leads to power gain in detecting enrichment between the ChIP and the control samples. Our findings have important implications for multiplexing samples.

## Introduction

Control experiments such as input DNA (whole cell extract) or ChIP with a non-specific antibody (e.g., IgG anti-serum) are commonly used to estimate background read distribution and are shown to be critical for identifying enriched regions in ChIP-seq experiments [1]–[4]. Standard ChIP-seq studies utilize paired controls by dividing the sample of interest into two parts, one of which is ChIPed with the antibody of interest and the other is used as the control sample. This practice typically limits the sequencing depths of control samples since resources are divided between ChIP and control samples and investigators are left wondering whether they have sufficient genomic coverage for their control samples. The issue of resources allocated to control samples remain highly significant in large scale ChIP-seq studies with 10s or 100s of diseased and non-diseased ChIPed individuals [5].

In [1], authors observed identification of more peaks when a ChIP-seq dataset was normalized against a more deeply sequenced input dataset. In a recent analysis [6], deeper sequencing of the input sample led to better detection specificity. The recent ENCODE ChIP-seq guidelines [7] suggest that the sequencing depth of the control experiment should be at least equal to and preferably larger than that of the ChIP sample.

We investigate *in silico* pooling designs, powered by the recent developments in sequencing technology, e.g., HiSeq 2000 of Illumina and multiplexing, for individually sequenced control samples. Although pooling of control samples generated within the same chromatin preparation step is commonly practiced, our investigation goes beyond this simple setting and inquires whether *in silico* pooling is a feasible option for input samples coming from different chromatin preparation steps. First, we establish that Pearson correlation between control samples within multiple biological replicates, multiple treatment conditions, and multiple cell/tissue types exhibit strong bi-modality indicating two classes, i.e., high and low, of within group correlations. Then, we show with multiple ChIP-seq experiments that *in silico* pooling designs increase the coverage for the control samples and lead to substantial power gain for the analysis of the ChIP-seq experiments within the high correlation class. Our computational experiments encompass datasets of wide range of genomic coverage from multiple organisms.

## Materials and Methods

### Mapped read files

We utilize a wide variety of datasets from worm *C. elegans*, rat, and human. Table 1 provides a list of these datasets.

**Table 1:** Description of the three classes of samples for *in silico* pooling analysis.

	Same treatment condition	Same tissue or cell line	Dataset
Multiple Biological Replicates Group (MBRG)	YES	YES	*C. elegans* Pha-4 (2 replicates) Rat Sox10 (2 replicates) *Hdac8 (K562) (2 replicates) Rat H3K27ac (2 replicates)
Multiple Treatments Group (MTG)	NO	YES	*c-Jun (K562) with IFN-Gamma treatment
Multiple Cell Lines Group (MCG)	YES	NO	*c-Fos (K562 vs. Huvec) *CTCF (Hct116 vs. Hpaf)

These three groups were evaluated separately for the feasibility and advantages of *in silico* pooling.

*: ENCODE dataset.

#### ENCODE datasets

The publicly available human datasets are from four different groups participating in the ENCODE consortia [8]. These datasets consist of input control samples from biological replicates (with different chromatin preparation steps), multiple treatments within same cell line, and multiple cell lines. The sequencing depths of the samples vary between 1.8 and 46 million (M) reads. A detailed list of these datasets is available as Table S1 in File S1. We use these datasets for investigating similarities between input samples and focus on a small subset to evaluate the impact of *in silico* pooling on peak detection. We investigate *in silico* pooling on three classes of samples (Table 1). The first class, named **M**ultiple **B**iological **R**eplicates **G**roup (MBRG), considers biological replicates that share same treatment or cellular conditions. The second class is **M**ultiple **T**reatments **G**roup (MTG) and encompasses samples that are from same type of cell lines or tissues but have undergone different treatments. The third class, **M**ultiple **C**ell Lines **G**roup (MCG) represents the most heterogeneous group and consists of samples from different cell lines and tissues but with similar treatment conditions. ENCODE input control samples have multiple samples in each of these three categories. We use c-Jun input samples from the K562 cell line profiled before and 30 minutes and 6 hours after stimulation with IFN-gamma as an example of MTG. The number of uniquely mapping reads for these experiments vary between 13 M and 18 M. As an example of MCG, we use c-Fos input samples from K562 and Huvec cell lines both of which were generated at UC Davis. In addition, we used a c-Fos sample from Huvec cell line that was generated at Yale University to investigate the lab effect on pooling. For these samples, the number of uniquely mapping reads are 29 M, 20 M, and 45 M, respectively. As another example of MCG, we consider CTCF profiling in Hct116 and Hpaf cell lines. These samples have 21 M and 20 M uniquely mapping reads, respectively. In addition, we also utilize two replicates of Hdac8 profiling in K562 cell lines as an example of MBRG. In short, these experiments encompass multiple ENCODE samples in each of the three classes of samples that we are interested in evaluating *in silico* pooling for.

#### *C. elegans* Pha-4 dataset

This modENCODE dataset [9] profiles transcription factor Pha-4 in the first stage of *C. elegans* larval development (L1) under starvation conditions with matching input controls. The number of uniquely mapping reads of two biological replicate controls are 11 M and 15 M. These high coverage experiments represent an example from the MBRG.

#### Rat Sox10 dataset

This dataset is also an example of MBRG and profiles Sox10 in spinal cords of two biological replicate rats. The numbers of uniquely mapping reads for the controls are 27 M and 88 M, respectively.

#### Rat H3K27ac dataset

This dataset is also an example of MBRG and profiles acethylation of H3K27 (H3K27ac), which commonly marks enhancers [10] and exhibits broader peak patterns compared to TFs, in peripheral nerves of two biological replicate rats. The numbers of uniquely mapping reads for the controls are 23 M and 26 M, respectively.

### Similarity calculation

In order to define a similarity measure between any two control samples, we first binned the genome into 200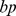 intervals and counted the number of reads within each bin after reads are extended to the average fragment length. Then, for the ENCODE samples, we consider the ENCODE reported peak lists (downloaded from hgdownload.cse.ucsc.edu) for which these input samples were used as control across multiple ChIP experiments and obtain a master list of ChIP-enriched regions. We use this master list to generate a read count vector across bins spanning these enriched regions for each sample. Dissimilarity between any two samples is based on one minus the Pearson correlation between their read count vectors. Hierarchical clustering of the samples based on normalized Euclidean distance between sample read count vectors is available as Figure S4 in File S1. For the other datasets, we first identify ChIP-enriched regions using MOSAiCS [11] and follow the above procedure with these peak lists. As a result, the reported Pearson correlations are between bin-level read count vectors spanning ChIP-enriched regions of the genomes.

### *In silico* pooling

For all the computational experiments, we randomly sample from the set of uniquely mapping reads to generate datasets of lower depths. *In silico* pooling simply combines the reads of different samples. We repeat all the pooling experiments five times to assess variability. The sub-sampling proportions vary for different datasets. For the *C. elegans* Pha-4 controls, we first randomly sub-sample 0.5%, 5%, 25%, and 50% of each of the biological matching control sample, generating a total of 8 control samples. We then treat these as multiplexed samples representing different coverage. We generate *in silico* pooling samples from these by pooling two sub-samples with similar depth between the biological replicates. This results in four *in silico* pooled samples with depths equivalent to 1%, 10%, 50%, and 100% of the full matching input sample, which we refer as the gold standard input sample.

For the rat Sox10 dataset, we use three types of *in silico* pooling and three types of gold standard inputs to accommodate the significant difference in the sequencing depths of the two biological replicates (Table 2). Let 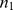 and 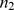 denote the depths of the two replicates with 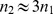. The first scheme pools half of each sample and uses an equal number of reads from replicate two as the gold standard. The second scheme pools *n*_1_/2 reads from each of the replicates and compares with the gold standard of 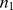 reads from the second sample. The third scheme pools 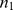 reads from each sample and compares with 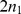 reads from the second replicate as the gold standard.

**Table 2:** Summary of the in silico pooling experiments for the Sox10 samples.

	# of reads from R1	# of reads from R2	Gold Standard
Pool 1	*n*_1_/2	*n*_2_/2	*n*_1_/2+*n*_2_/2 from R2 (GS_1)
Pool 2	*n*_1_/2	*n*_1_/2	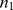 from R2 (GS_2)
Pool 3	*n*_1_	*n*_1_	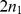 from R2 (GS_3)

R1 and R2 denote the two biological replicates.

For the c-Jun dataset, we have four input samples with similar depths: two for the untreated sample and one for each of the two IFN-gamma stimulated samples. Therefore, we randomly sample a quarter from each of the input samples across three treatments for *in silico* pooling. For H3K27ac, c-Fos, and the other ENCODE datasets, we randomly sample half of the reads from each input sample for pooling.

### Comparison between peak sets obtained with different control input samples

We perform all the peak identification with the two-sample MOSAiCS [11] model that compares the ChIP and the input samples. We illustrate the robustness of the results to the peak caller used by utilizing another commonly used peak caller, SPP [12] (Figure S1 in File S1). All the MOSAiCS fits are tuned so that the goodness-of-fit (GOF) plots exhibit good overall fits. We analyze each individual ChIP sample with its corresponding full size matching input sample to generate gold standard peak sets. We denote these peak sets by 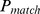, the peak sets obtained by lower depth matching input by 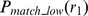, and peak sets obtained by *in silico* pooling by 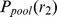. Here, the depths of the lower depth and pooling samples are indexed by 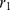 and 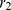, where they each have 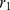 and 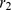 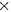 (# of reads in the full matching input) reads, respectively. Our comparisons use 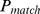 as the gold standard and compare the performances of 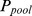 and 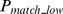. The overall design, unless specified otherwise, for 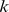 samples is that 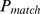 is obtained by using full matching input control sample of depth 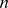, for each of the 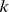 samples, 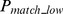 is by matching input control of depth *n*/*k* for each of the samples, and 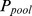 is by combining these 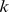 matching input controls to result in a pooled sample of depth 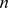, i.e., 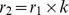. The evaluations are performed by comparing 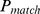, 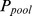, and 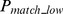 for each individual ChIP sample and the overlap proportions, i.e., sensitivity, are averaged within each dataset. For example, for the rat Sox10 dataset, we perform a comparison between the three peaks sets for each of the two ChIP samples and report average. In addition, since each pooling experiment is repeated 5 times, the overall number of comparisons that the reported overlap proportions depend on vary from 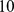 to 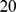.

For the Sox10 dataset, we further use positive predictive value to compare the performances of 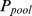 and 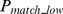 with 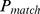 (Figure S3(b) in File S1). In addition, we evaluate the consensus motif occurrence rates for the 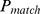, 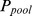, and 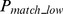 peak sets of sequence specific TF Pha-4. Specifically, we use matrix scan utility of Regulatory Sequence Analysis Tools [13] with a p-value threshold of 0.01 to scan the peaks with the Pha-4 position specific weight matrix from JASPAR [14] (MA0546.1) (Figure S2 in File S1) and assess whether similar proportions of the 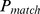, 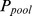, and 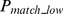 peaks have at least one motif.

## Results

### Clustering of multiple input samples from the ENCODE project

**Figure 1:**
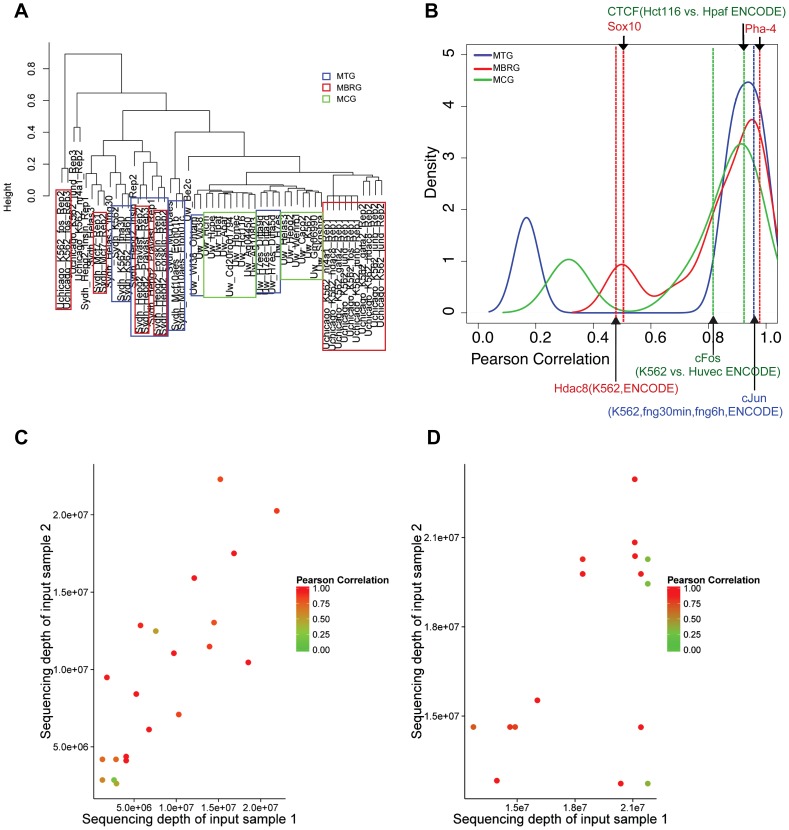
Exploratory analysis of ENCODE input samples. (a) Hierarchical clustering of 53 input samples from the ENCODE project. (b) Empirical density of the Pearson correlations within Multiple Biological Replicates (MBRG), Multiple Treatments (MTG), and Multiple Cell lines (MCG) Groups. (c) Effect of sequencing depth on the correlations within MBRG and MTG. (d) Effect of sequencing depth on the correlations within MCG.

We hierarchically clustered 53 input control samples from the ENCODE project (Figure 1(a)) based on their bin-level input read count vectors over the master list of ChIP-enriched regions. Figure 1(a) reveals a strong lab effect where a few number of similar experiments (same cell type) performed by different labs cluster separately than their group. For example, although a few of the UChicago K562 inputs cluster with Snyder K562 input samples, majority of them cluster with the rest of the UChicago input samples. However, within each lab, biological replicates tend to cluster together as opposed to multiple treatments or cell line/tissue types with a few exceptions. Hepg2 input sample ("Sydh_hepg2_Pravast_rep1"), which has a much lower sequencing depth than the other two replicate samples of the same condition, cluster slightly more closely with another set of Hepg2 samples than these two biological replicates. The low sequencing depth of this sample is a likely explanation for this result. Although Pearson correlation is a scale-free measurement of similarity, it is affected by sequencing depths of samples, as is illustrated below and Figure 1(c). This clustering reveals a higher overall similarity within MBRG compared to MTG and MCG. Hierarchical clustering based on normalized Euclidean distance leads to the same conclusions (Figure S4 in File S1).

Next, we set out to assess the distribution of Pearson correlations within each of the biological replicates, treatments, and cell lines groups. Figure 1(b) depicts the empirical density of the Pearson correlations within each group. We observe a bi-modal density within each group and the samples can be divided into two classes by using a Pearson correlation threshold of 0.6 to 0.8. These bi-modal densities reveal that within each of MBRG, MTG, and MCG, there are pairs of samples with both *high* and *low* Pearson correlations. The median correlation of the low class for MBRG is higher than those of MTG and MCG, confirming the higher level of overall similarity for MBRG. We also investigate the effect of the sequencing depths on the correlation between samples. Figure 1(c) illustrates that when the sequencing depths are low, the correlations between samples that are biological replicates or are exposed to different treatments on the same cell/tissue types are lower than the correlations observed at higher sequencing depths. However, for samples from more diverse conditions, i.e., different cell types, low correlations arise irrespective of sequencing depths (Figure 1(d)).

### *In silico* pooling within MBRG: *C. elegans* Pha-4 dataset

**Figure 2:**
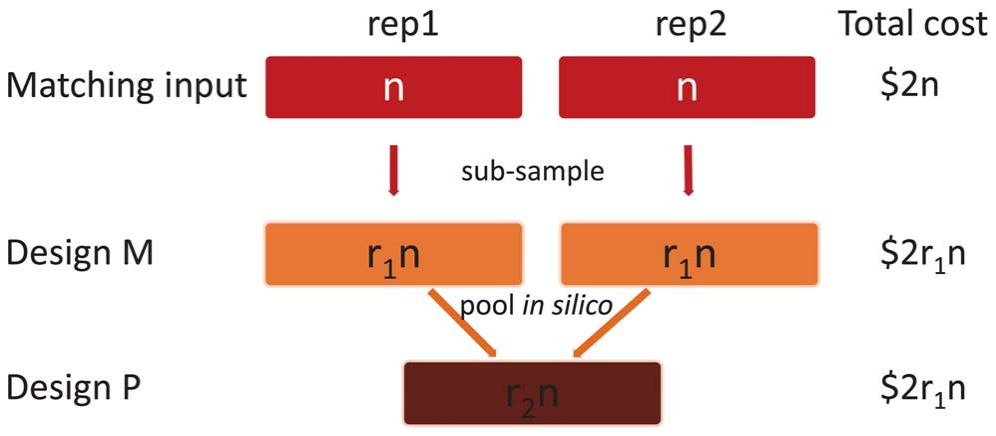
Matching (M) and *in silico* pooling (P) designs for 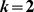 multiplexed samples. Designs M and P have the same cost since Design P simply pools reads from Design M *in silico* (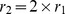).

**Figure 3:**
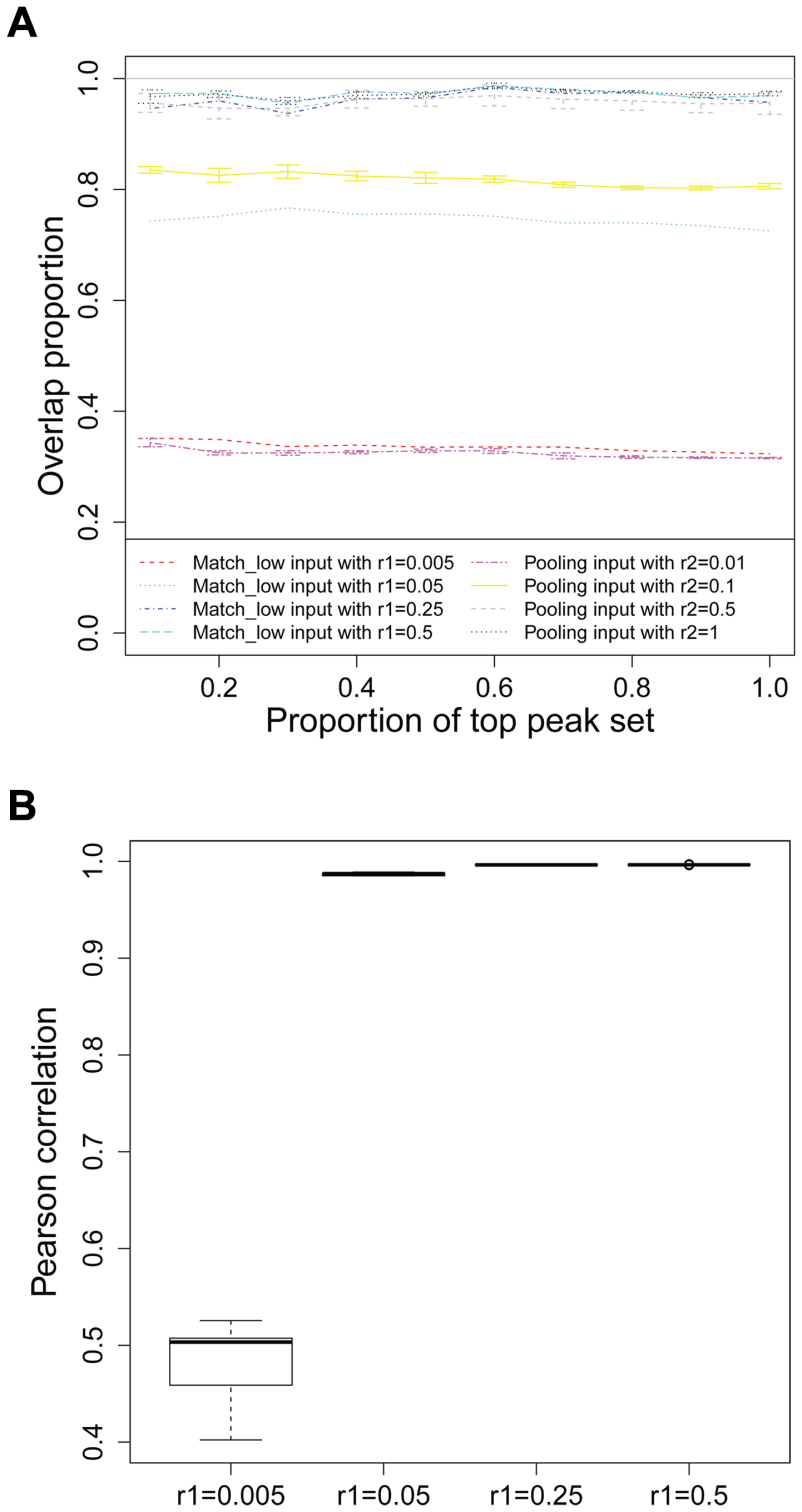
Evaluation of *in silico* pooling within MBRG with C. elegans Pha-4 experiments. (a) Overlap comparisons between 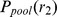 and 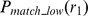 vs. 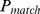; (b) Correlations between sub-sampled inputs.

We use the deeply sequenced *C. elegans* Pha-4 experiments from modENCODE [15] for an in depth analysis of *in silico* pooling between two biological replicates. Our computational experiments are aimed at deciphering when *in silico* pooling is an advantageous strategy for ChIP-seq experiments over multiple experimental units, e.g., people, strains, animals, under same treatment and cellular conditions. We specifically compare two types of designs as illustrated in Figure 2. In design M, the replicates are multiplexed to generate matching input samples of size 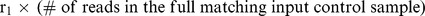 reads for each of two biological replicates. We varied 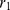 as 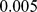, 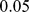, 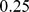, and 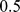. 0.5 M reads in *C. elegans* correspond to 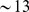 M reads on the mappable human genome and 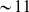 M reads on the mappable rat genome; hence 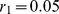 roughly corresponds to 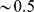 M *C. elegans* reads and 13 M human reads. Then, each ChIP sample is analyzed with respect to its corresponding matching input sample which is smaller in size than the gold standard matching input sample. In design P, the multiplexed control samples are pooled *in silico*, generating a control sample with two times the depth of the individual samples, and each ChIP sample is analyzed with respect to this pooled sample. Figure 3(a) compares peaks identified using each of these different depth control samples across the two designs with the gold standard 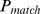 as a function of the top 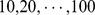
% of the peak set. Peaks within each set are ordered with respect to their ChIP enrichment posterior probabilities reported by MOSAiCS. We observe that when 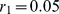 leading to 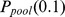, the overlap between 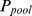 and 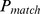 is greater than 0.8, and when 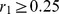, the overlap between 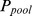 and 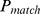 is approaching 1. This indicates that *in silico* pooling, which only sequences 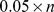 reads for every biological replicate and yields a total of 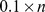 input control reads, generates the same set of ChIP-enriched regions as the matching input design where each biological replicate has 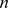 reads (a total 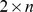 input control reads). This is roughly a factor of 20 decrease in the overall cost of sequencing the input library for these set of experiments.

We observe the most significant improvement due to *in silico* pooling when the replicate control samples have depths as small as 0.1/2-th of the full matching control sample. At lower depth, neither 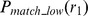 nor 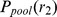 is able to capture 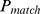; however, 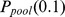 performs significantly better than 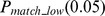 indicating that *in silico* pooling is able to identify peaks in the same rank order as the 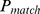.

For lower depth with 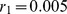, 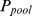 and 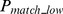 perform comparably, indicating that even for cases when pooling does not lead to improvements, it does not deteriorate the performance. For higher depths, 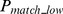 and 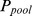 perform very similarly to 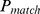 indicating that these depths have reached saturation in terms of information usable for estimating background read distribution of the ChIP sample. Figure S2 in File S1 illustrates that with 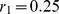, 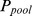 and 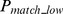 perform similarly to 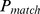 in terms of Pha-4 consensus motif occurrence rate. Next, we investigate the Pearson correlations between the input controls of biological replicates for varying 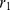. Figure 3(b) summarizes pairwise correlations between the replicates for different 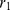 values. The correlation pattern exhibits a significant drop for 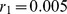 indicating that pooling is not likely to increase the effective depth when individual input control samples are only 0.5% of the full matching input control sample. The value of the Pearson correlation between biological replicates at which *in silico* pooling provides significant improvement agrees well with the empirical cut-off we derive for dividing samples within the MBRG into two in Figure 1(b).

### *In silico* pooling within MBRG: Rat Sox10 dataset

**Figure 4:**
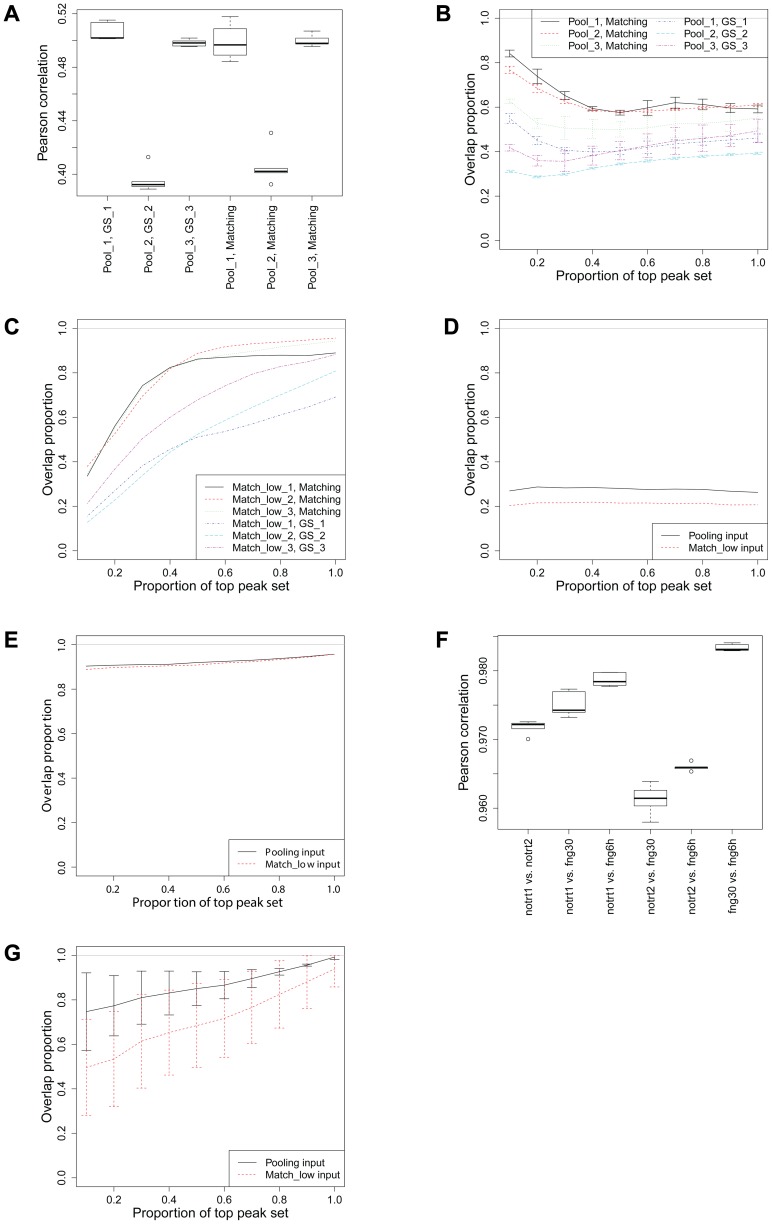
Evaluation of *in silico* pooling within MBRG (rat Sox10 and ENCODE Hdac8 datasets) and within MTG (c-Jun in K562 with three levels of treatment). (a) Sox10 correlations between two sub-sample inputs for all three pooling schemes based on peaks identified by using various gold standard input sets, where '(x, y)' refers to sub-sample inputs corresponding to pooling schemes x based on peaks by input y. (b) Sox10 overlap comparisons between 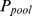 and 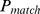, where '(x, y)' refers to comparison between 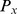 and 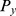. (c) Sox10 overlap comparisons between 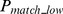 and 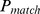. (d) Hdac8 overlap comparisons between 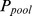 and 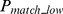 vs. 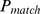. (e) H3K27ac overlap comparisons between 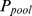 and 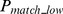 vs. 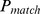. (f) c-Jun correlations between four sub-sample inputs. (g) c-Jun overlap comparisons between 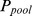 and 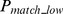 vs. 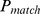.

Because the two replicates have significantly different sequencing depths for the Sox10 experiments, we generate gold standard matching input controls based on the replicate with the larger depth (Table 2) (denoted as GS_1, GS_2, GS_3 in Figures 4(a), (b), and (c)) and use the ChIP sample from this replicate for evaluations. Results based on the actual full depth matching inputs (denoted as Matching) are also presented for comparison. Figure 4(a) displays correlations between the sub-sampled inputs based on peaks identified by using various gold standard input sets. For example, (pool_1, GS_1) denotes correlations between an input sample of size *n*_1_/2 reads from replicate 1 and another input sample of size *n*_2_/2 reads from replicate 2. The correlations are based on bin-level data spanning top 10% of the ChIP-enriched regions that are identified when the ChIP replicate 2 is analyzed with its matching input of size *n*_1_/2+*n*_2_/2 reads from input replicate 2 (denoted as GS_1). Similarly, (pool_1, matching_1) denotes correlations between an input sample of size *n*_1_/2 reads from replicate 1 and another input sample of size *n*_2_/2 reads from replicate 2 where the ChIP-enriched regions are defined by analyzing both ChIP replicates with their original full matching input samples. Figure 4(a) illustrates that regardless of the pooling design and the gold standard used for the matching input sample, the correlations between sub-sampled input samples are lower than the empirical 0.6 to 0.8 threshold that partitions the Pearson correlations from the ENCODE samples into two groups. The effect of these low correlations is imminent on the comparison of 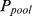 with 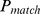 for all the three pooling schemes with different gold standards. Figure 4(b) compares peaks from the three pooling schemes to peaks from two types of matching inputs and reveals that none of the pooling schemes perform as well as the corresponding gold standard or matching input 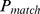. We also compare 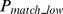 of replicate 2 corresponding to three pooling approaches with peaks from different gold standards (Figure 4(c)), e.g., Match_low_3 has 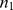 reads from replicate 2 as opposed to 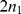 reads in GS_3 and 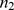 reads in Matching. The comparison between Figures 4(b) and 4(c) indicate that 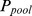 still performs better than 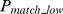 in terms of identifying peaks in the same rank order as in 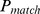 for the top 20% of the peaks. However, the overall sensitivity of 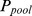 is lower than 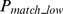 since the number of peaks including true positives and false positives in 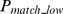 is significantly higher than in 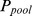 (Figure S3(a) in File S1). Figure S3(b) in File S1 compares the positive predictive values between 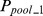 and 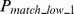 to the corresponding 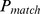, as well as 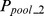 and 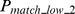 to the corresponding 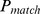. Neither of them have high positive predictive values and 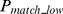 is slightly below 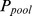 due to too many false positives. Therefore, when the correlation between two replicates is lower than the empirical 0.6 to 0.8 threshold, increasing the sequencing depths with further sequencing might be a better strategy.

### *In silico* pooling within MBRG: randomly selected ENCODE Hdac8 and histone H3K27ac datasets

We randomly pick one of the ENCODE datasets (Hdac8 profiling in K562) that exhibits low Pearson correlation across samples within the MBRG. The correlation between the two replicates of the Hdac8 dataset is only 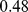, which classifies this sample into low correlation class in the empirical density of the Pearson correlations (Figure 1(b)). Figure 4(d) displays 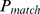, 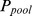, and 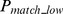 comparisons of these samples. Although, both 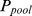 and 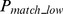 exhibit very little overlap with 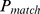, 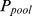 is significantly better than 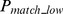. This suggests that if an experiment yields multiplexed samples of low depth, the optimal procedure is to increase the depth of each biological replicate input control sample; however, when this is not feasible, an analysis based on pooled control samples is better than using low depth matching input for each replicate.

As a final illustration for MBGR, we use the rat H3K27ac ChIP-seq dataset for two biological replicates. Figure 4(e) displays the comparisons of 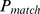, 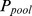, and 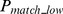. 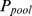 and 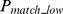 perform almost as good as 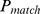 since the Pearson correlation between sub-sampled input samples is extremely high (0.99).

### *In silico* pooling within MTG: ENCODE c-Jun (K562) dataset

The sub-sampled inputs of the c-Jun dataset exhibit very high correlations despite their low sequencing depths (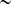 20 M mappable reads, Figure 4(f)). Figure 4(g) compares 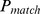 with 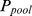 and 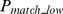 and reports averages of overlapping proportions when each of the three ChIP samples representing different treatments are analyzed with respect to matching, pooling, low depth matching input samples. The overlap performances agree well with the observed high correlations. 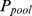 compares well with 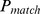 and is significantly better and less variable than 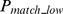.

### *In silico* pooling within MCG: ENCODE c-Fos (K562 vs. Huvec) and CTCF (Hct116 vs. Hpaf) datasets

**Figure 5:**
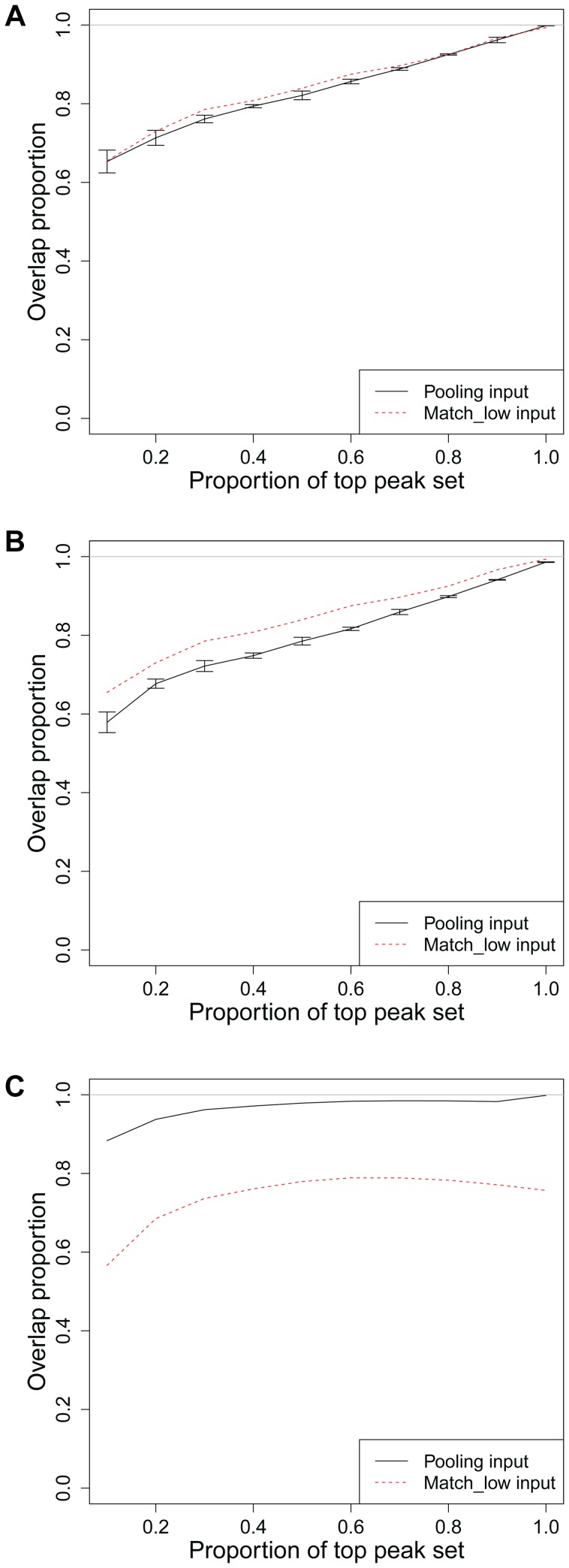
Evaluation of *in silico* pooling within MCG with ENCODE samples. a) c-Fos overlap comparisons between 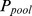 and 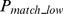 vs. 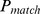 in cell lines K562 vs. Huvec. (b) c-Fos overlap comparisons between 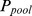 (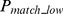) and 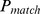 in cell line Huvec with inputs from two different labs. (c) CTCF overlap comparisons between 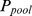 and 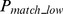 vs. 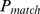 in cell lines Hct116 and Hpaf.

Figure 5(a) reports 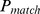, 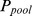, and 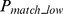 comparisons of the c-Fos analysis in K562 and Huvec cell lines. The reported overlap proportions are averages of the analysis of the K562 and Huvec c-Fos ChIP samples with varying input controls. Both 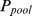 and 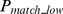 have rankings of the enriched regions different than that of 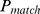; however, both controls are able to identify enriched regions captured by 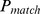. The correlations between the two sub-sampled inputs vary around 0.83 across different sub-sampling experiments and support pooling. However, as illustrated in Figure 5(a), both the individual matching sub-sample inputs and the *in silico* pooling inputs perform equally well. Since c-Fos is profiled in Huvec cells by two different labs, we also evaluated the possibility of pooling input samples generated by different labs. The correlations between low depth input samples generated from the input samples of two different labs varied around 0.82 across different sub-sampling experiments. Figure 5(b) illustrates that even though both 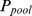, obtained from combining input samples from two labs, and 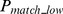 are able to identify peaks of 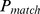, there is a significant difference between 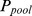 and 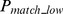 in terms of the ranking of the peaks. In particular, pooling input samples across different labs seems to generate a worse ranking than that of using a low depth matching input.

As another example of *in silico* pooling within MCG, we randomly picked the CTCF ChIP-seq datasets from Hct116 and Hpaf cells from the set of MCG samples with high Pearson correlation, e.g., correlation between the full matching input samples of these cells is 0.93. Figure 5(c) displays the comparisons of 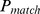, 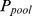, and 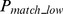. 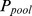 which has twice the depth of 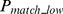 performs much better than 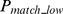 and is almost as good as 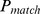.

## Discussion

We investigated the effect of *in silico* pooling of control samples within MBRG, MTG, and MCG across multiple datasets with varying levels of genomic coverage from multiple organisms. Our computational experiment based on vast amounts of ENCODE data show that pairwise Pearson correlations between control samples originating from MBRG, MTG, and MCG exhibit a strong bi-modality indicating two classes representing whether or not *in silico* pooling may lead to power gain in detecting enrichment between the ChIP and the control samples. We demonstrate that *in silico* pooling designs increase the coverage of control samples and perform as well as the matching input within the high correlation class using multiple ChIP-seq experiments. Comparison of the input samples with simple Pearson correlation across candidate enrichment regions provided consistent results across the large numbers of datasets we have considered. Although we considered more formal testing approaches (e.g., differential enrichment analysis of the input samples with DBChIP [16] and irreducible discovery rate (IDR) [17]) for comparing two or more input samples, the overall insufficient depths of the input samples (median depths ranged between 0 to 28 for the *C. elegans* samples; 2 to 4 for the rat Sox10 samples, and is 2 for the ENCODE c-Fos samples) resulted in discrete p-values with many ties and prohibited robust application of these approaches.

Our findings have important implications for multiplexing control samples in ChIP-seq experiments, especially for samples from MBRG. Specifically, they endorse starting with relatively low coverage of multiplexed control samples and calculating their pairwise Pearson correlations for candidate enrichment regions that may be obtained by an initial comparison of ChIP and control samples. If the pairwise correlations belong to the empirical high correlation class, multiplexed samples can be pooled as the *in silico* pooling input and used for peak detection. Otherwise, increasing the coverage of control samples by further sequencing is essential to gain power in detecting enrichment between the ChIP and the control samples. Although the multiplexing control samples with relatively low coverage are only available control samples for candidate enrichment region selection, our experiments based on *C. elegans* Pha-4 and ENCODE Hdac8 data show that the pairwise input sample correlations are very comparable for candidate enrichment regions that are obtained by full matching input and lower depth inputs.

## Supporting Information

File S1 **Supporting figures and tables.**
(PDF)
