# Supplementary material for: In Silico Pooling of ChIP-seq Control Experiments
Source: PLoS One. 2014 Nov 7;9(11):e109691. doi: 10.1371/journal.pone.0109691 (PMC4224375; doi:10.1371/journal.pone.0109691)
Supplement: File S1 — Supporting figures and tables. (PDF) [file pone.0109691.s001.pdf]

Supplementary information for "*In silico* pooling of ChIP-seq control experiments"

In silico pooling within MBRG: modENCODE *C. elegans* Pha-4 dataset

This section illustrates *in silico* pooling within MBRG based on the deeply sequenced modENCODE dataset Pha-4 using another commonly used peak caller SPP. We randomly sub-sampled 0.05%, 5%, 25%, and 50% of each of the biological matching control samples and treated them as multiplexed samples representing different coverages. Then, we generated *in silico* pooling samples by pooling two sub-samples with similar depths across the biological replicates, which is similar to the *in silico* pooling procedure based on MOSAiCS. The sub-sampled inputs of *C. elegans* dataset exhibited high correlations that were larger than the empirical 0.6 to 0.8 cut-off (Figure S1(b)) when r_1 was greater than 0.005. Figure S1(a) compares $P_{match\_low}(r_1)$ and $P_{pool}(r_2)$, where r_1 is 0.005, 0.05, 0.25, and 0.5, and r_2 is 0.01, 0.1, 0.5, and 1, respectively, for ChIP replicate 1. Comparisons of the averages of overlap proportions across different sub-sampling experiments indicate that both P_{match_low} and P_{pool} perform comparably well when r_1 is greater than 0.05. P_{pool} is always larger than 0.8 and significantly better than P_{match_low} at the level of sequencing depth with $r_1 = 0.05$. Neither P_{match_low} nor P_{pool} exhibit high enough overlap proportions at $r_1 = 0.005$ with extremely low correlation between the sub-sampled inputs. The overall performance of *in silico* pooling samples based on SPP is similar to their performances based on MOSAiCS.

(a) (b)

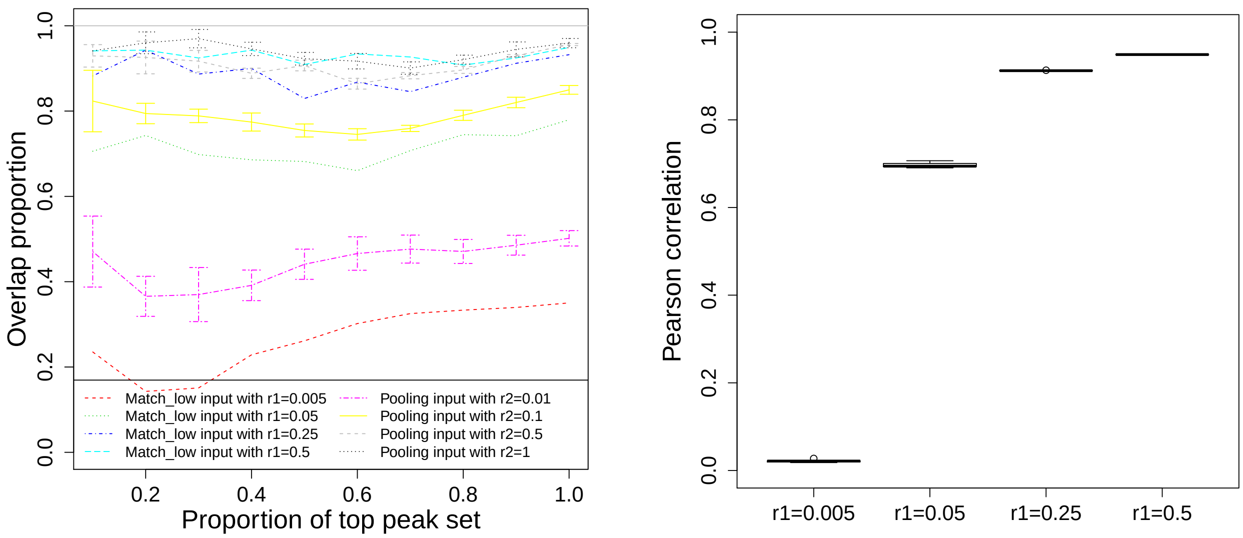

Figure S1: *Evaluation of in silico pooling with C. elegans Pha-4 experiments.* (a) Overlap comparisons between $P_{pool}(r_2)$ and $P_{match\_low}(r_1)$ vs. P_{match}; (b) Correlations between two sub-sampled inputs.

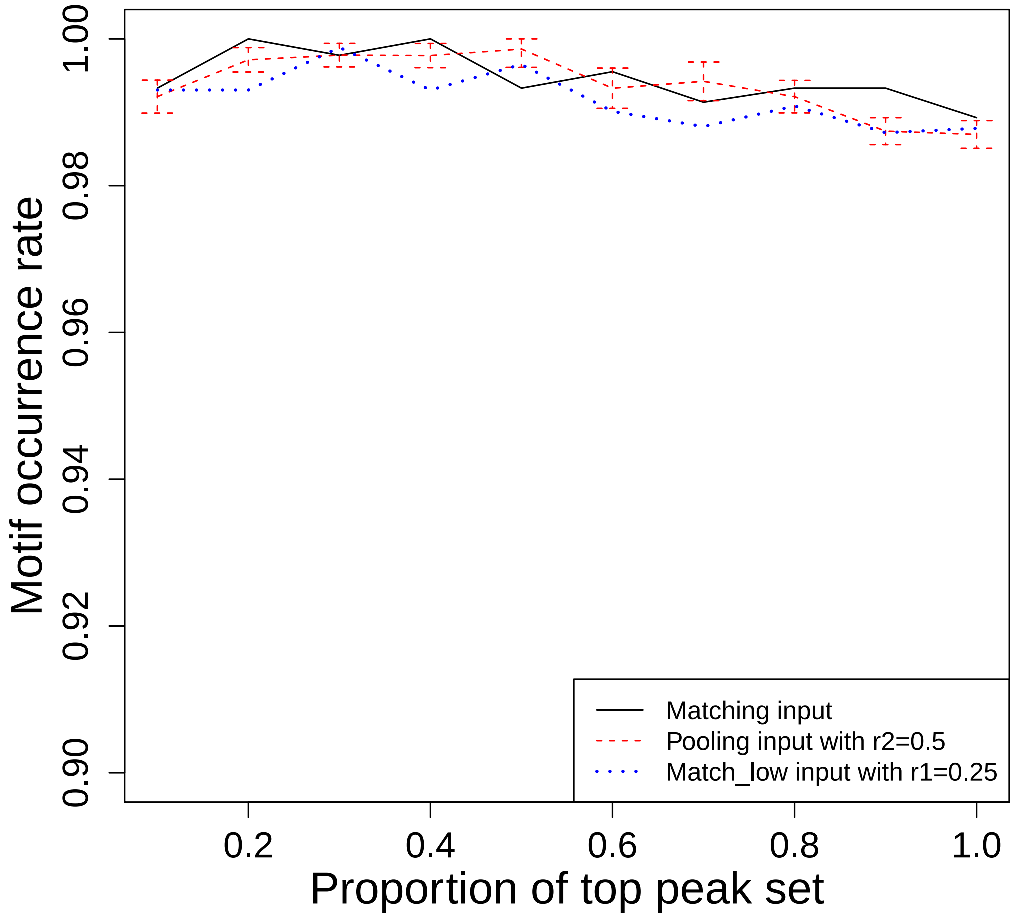

Figure S2: *Pha-4 motif occurrence rates of the* P_{pool}, P_{match_low}, *and* P_{match} *peak sets for the C. elegans dataset.*

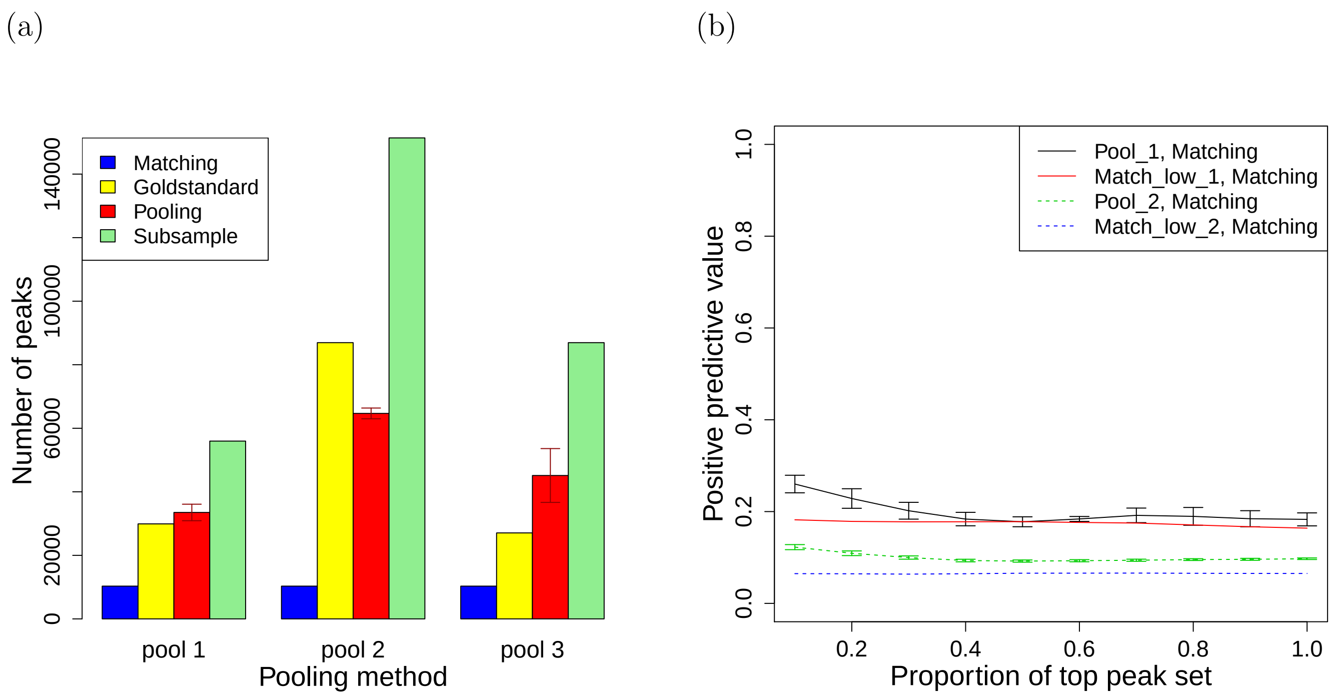

Figure S3: *Evaluation of in silico pooling with the Sox10 experiments.* (a) Numbers of Sox10 peaks based on different input samples. For each pooling strategy, the corresponding matching and gold-standard input are also reported. (b) *Positive predictive values of* P_{pool} *and* P_{match_low} *for (pool1, matching) and (pool2, matching) for the Sox10 dataset.*

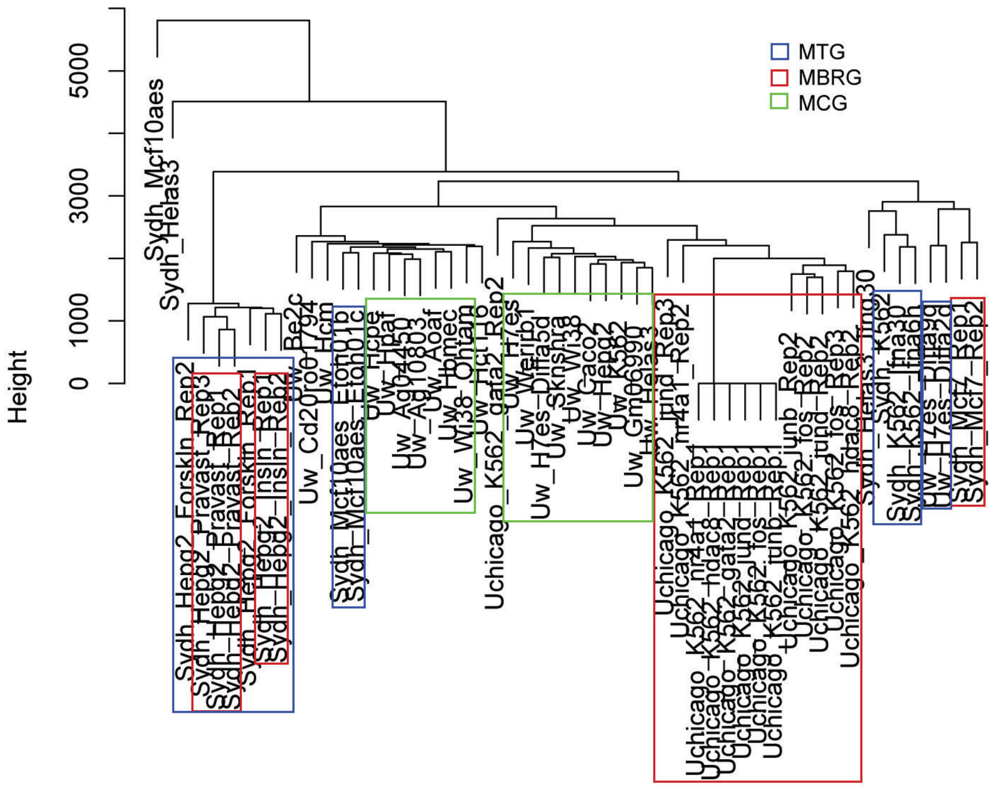

Figure S4: *Hierarchical clustering based on normalized Euclidean distance.*

ENCODE sample name	# of uniquely mapping reads
Sydh_Helas3_Ifng30	17,004,373
Sydh_Helas3	21,514,332
Sydh_Hepg2_Forskln_Rep1	4,062,995
Sydh_Hepg2_Forskln_Rep2	4,375,166
Sydh_Hepg2_Insln_Rep1	2,916,974
Sydh_Hepg2_Insln_Rep2	2,629,722
Sydh_Hepg2_Pravast_Rep1	1,255,003
Sydh_Hepg2_Pravast_Rep2	2,859,141
Sydh_Hepg2_Pravast_Rep3	4,191,240
Sydh_K562_Ifna30	16,846,771
Sydh_K562_Ifna6h	17,505,356
Sydh_Mcf10aes_Etoh01b	21,911,540
Sydh_Mcf10aes_Etoh01c	20,254,301
Sydh_Mcf10aes	46,127,579
Sydh_Mcf7_Rep1	12,128,604
Sydh_Mcf7_Rep2	15,911,675
Uchicago_K562_fos_Rep1	NA
Uchicago_K562_fos_Rep2	13,943,070
Uchicago_K562_fos_Rep3	11,485,309
Uchicago_K562_junb_Rep1	NA
Uchicago_K562_junb_Rep2	13,808,824
Uchicago_K562_jund_Rep1	NA
Uchicago_K562_jund_Rep2	13,034,962
Uchicago_K562_jund_Rep3	17,076,080
Uchicago_K562_gata2_Rep1	NA
Uchicago_K562_gata2_Rep2	22,298,201
Uchicago_K562_hdac8_Rep1	NA
Uchicago_K562_hdac8_Rep2	NA
Uchicago_K562_nr4a1_Rep1	NA
Uchicago_K562_nr4a1_Rep2	20,212,833
Uw_Ag04450	18,400,427
Uw_H7es_Diffa2d	NA
Uw_H7es_Diffa5d	NA
Uw_H7es_Diffa9d	NA
Uw_H7es	NA
Uw_Ag10803	19,776,716
Uw_Aoaf	21,424,353
Uw_Hbmec	21,100,439
Uw_Be2c	NA
Uw_Hcm	22,959,759
Uw_Cd20ro01794	NA
Uw_Hcpe	20,835,811

Sample name	# of uniquely mapping reads
Uw_Wi38_Ohtam	NA
Uw_Wi38	NA
Uw_Hct116	21,115,507
Uw_Hpaf	20,376,309
Uw_Caco2	13,945,822
Uw_Gm06990	12,829,501
Uw_Helas3	14,897,699
Uw_Hepg2	14,631,322
Uw_K562	14,627,599
Uw_Sknshra	16,049,874
Uw_Werirb1	15,527,376
Broad_Gm12878_Rep1	6,768,855
Broad_Gm12878_Rep2	6,119,025
Broad_H1hesc_Rep1	10,294,975
Broad_H1hesc_Rep2	7,088,917
Broad_Hepg2_Rep1	1,784,520
Broad_Hepg2_Rep2	9,487,898
Broad_Hmec_Rep1	5,763,689
Broad_Hmec_Rep2	12,845,159
Broad_Hsmm_Rep1	9,721,907
Broad_Hsmm_Rep2	11,052,841
Broad_Huvec_Rep1	7,574,087
Broad_Huvec_Rep2	12,484,829
Broad_K562_Rep1	18,534,334
Broad_K562_Rep2	NA
Braod_Nhek_Rep1	11,205,898
Braod_Nhek_Rep2	9,921,760
Broad_Nhlf_Rep1	5,258,419
Broad_Nhlf_Rep2	8,415,815

Table S1: *List of ENCODE datasets from four different groups.* Numbers of uniquely mapping reads are obtained from summary (http://genome.ucsc.edu/ENCODE/qualityMetrics.html). "NA" indicates missing information in the summary.
